# A Model for Consolidating High-Risk Allergy Procedures in Clinic

**DOI:** 10.31486/toj.24.0085

**Published:** 2024

**Authors:** Monica Hajirawala, Amber Hardeman, Nina Hein, John C. Carlson

**Affiliations:** ^1^ Department of Allergy and Immunology, University of South Florida Health, Tampa, FL; ^2^ Department of Allergy and Immunology, Tulane University School of Medicine, New Orleans, LA; ^3^ Department of Allergy and Immunology, Ochsner Clinic Foundation, New Orleans, LA

**Keywords:** *Allergy and immunology*, *anaphylaxis*, *desensitization–immunologic*, *hypersensitivity*, *skin tests*

## Abstract

**Background:** Allergists perform a range of procedures with inherent risks of anaphylaxis. This study developed risk assessments for various procedures performed at our specialized referral center based on the frequency of epinephrine use during these procedures.

**Methods:** During a 5.5-year period, 5 allergists referred patients to a monthly high-risk procedure clinic (total of 66 clinic days). We conducted a retrospective medical records review from 2016 to 2021 to assess the types of procedures performed, instances of procedure termination, and use of epinephrine.

**Results:** A total of 596 procedures were performed: 305 food challenges, 103 aeroallergen immunotherapy rush inductions, 75 drug challenges, 66 ultrarush inductions of venom immunotherapy, 12 drug desensitizations, 14 vaccine challenges (11 COVID-19 [coronavirus disease 2019], 2 influenza, 1 Tdap [tetanus, diphtheria, and pertussis]), and 21 miscellaneous nonvaccine challenges. Most procedures (n=551, 92.4%) were completed; 45 procedures (7.6%) were aborted early because of patient, parent, or physician requests. Reasons included the child not wanting to eat the food, the patient developing a headache, and other factors. Fifty-one of the 596 procedures (8.6%) required epinephrine administration: 32/305 (10.5%) for food challenges, 12/103 (11.7%) for aeroallergen immunotherapy rush inductions, 2/75 (2.7%) for drug challenges, 2/66 (3.0%) for ultrarush inductions of venom immunotherapy, 3/12 (25.0%) for drug desensitizations, and 0/35 (0%) for other challenges. Two patients required emergency department transfers, with no instances resulting in hospitalization or patient mortality.

**Conclusion:** These data identify risks associated with diverse procedures conducted in allergy clinics. While 8.6% of cases required epinephrine, the majority of reactions were manageable within the clinic setting. These findings underscore the allergist's role in performing procedures with potential anaphylactic outcomes and managing anaphylaxis when it occurs in the clinic setting. Additionally, the procedure clinic model is an effective educational tool that provides fellows-in-training with exposure to the identification and management of acute anaphylaxis.

## INTRODUCTION

Allergists perform a wide range of procedures—ranging from challenges involving food, medications, and vaccines to rush and ultrarush inductions for aeroallergen and venom allergy, respectively—that have the inherent risk of inducing anaphylaxis. The word *rush* in aeroallergen immunotherapy rush induction refers to a protocol in which the induction buildup phase of immunotherapy is accelerated over several hours, allowing patients to reach maintenance doses quickly. After rush induction, patients require several additional weekly doses before reaching monthly maintenance. In contrast, *ultrarush* induction of venom immunotherapy is an even faster protocol in which the induction buildup phase is completed within hours and requires only one additional appointment the following week before transitioning to monthly maintenance. Such acceleration is often critical for managing high-risk venom-allergic patients who require rapid protection.

The effective management of anaphylaxis requires prompt evaluation and treatment. In addition to expertise in anaphylaxis identification and management, allergists also have experience in discerning psychosomatic reactions that can mimic symptoms of anaphylaxis, as anxiety can induce the sensation of throat constriction, nausea, flushing, and/or urticaria in patients.^1^

Because of the time required for assessing and addressing reactions, allergists often face the necessity of blocking clinic schedules during procedures. However, the published rates of reactions vary, not only across different types of procedures but also among studies investigating the same procedure.

Estimating the risk of reaction for oral food challenges is particularly difficult. Patient histories may range from cases with a history of multisystem anaphylaxis (high pretest probability) to cases with no prior reaction history and only elevated immunoglobulin E (IgE) (very low pretest probability). While the perception of food allergy prevalence may be elevated because of the subjectivity of clinical history, the true prevalence of food allergy is reported to be approximately 6% in children and 3% in adults.^2,3^ Graded oral food challenges are a cost- and time-efficient method for evaluating allergic reactivity to foods, particularly when clinical histories diverge from clear anaphylaxis.^2,4^ Oral food challenges are considered safe procedures, and most children who undergo them are able to reintroduce the food into their diets following the procedure. Notably, 2 studies involving 544 and 701 oral food challenges showed reaction rates between 18.8% and 48.3%, with epinephrine administration rates ranging from 1.7% to 2.4%.^3,5^

The landscape of drug challenge histories also introduces heterogenous risks, with drug allergies accounting for 5% to 10% of adverse drug reactions.^6^ Drug allergies can lead to treatment delays, result in suboptimal medication use, and increase morbidity.^7^ Interestingly, 94% of patients evaluated for penicillin allergy tolerated the medication without adverse reactions, although outcomes of challenges to other medications remain less well defined in the literature.^8^

Aeroallergen immunotherapy rush induction poses varied risks depending on specific protocols. One study had a reaction rate of 73% when the procedure was performed without premedications, but the reaction rate was 27% when patients were pretreated with H1 antagonists, H2 antagonists, and systemic corticosteroids.^9^ Reaction rates are highly variable across studies, although most systemic reactions in rush desensitization protocols are mild, such as flushing reactions.^10^ Pediatric patients receiving subcutaneous immunotherapy show a high rate of these milder systemic reactions compared to adults.^11^ In contrast, the rate of systemic reactions to ultrarush induction of venom immunotherapy is generally lower than the higher reaction rates seen in aeroallergen immunotherapy rush induction procedures. Systemic reactions to Hymenoptera venom occur in 0.5% to 3.3% of the US population.^12^ A Cochrane review of venom immunotherapy concluded that the risk of systemic reaction from venom immunotherapy is 2.7% compared to an untreated patient's 39.8% risk of developing a systemic allergic reaction to a sting.^13^ Ultrarush protocols for stinging Hymenoptera venom immunotherapy have been associated with a decreased risk for systemic reactions compared to conventional buildup,^14^ with rates of systemic adverse reactions reported in the Cochrane review of 14.2% of participants treated for bee venom allergy and 2.8% of participants treated for wasp venom allergy.^13^

We undertook this study to better understand the rates of reaction for procedures performed in our system's high-risk allergy procedure clinic.

## METHODS

At our medical center in New Orleans, Louisiana, patients requiring procedures associated with a perceived elevated risk of reaction are systematically directed to a high-risk clinic dedicated to performing these procedures. These referrals are made based on the judgment of the 5 referring allergists/immunologists in our system who work throughout the greater New Orleans area. The hospital-based clinic is overseen by a single allergist with 2 allergy/immunology fellows, supplemented by a varying number of residents and students.

Following approval by the Ochsner Health Institutional Review Board, we conducted a retrospective review of patients who presented to this high-risk clinic from 2016 to 2021. Cases were identified through an Epic (Epic Systems Corporation) query of patients seen on high-risk clinic days and did not include procedures performed by the same physicians on other days in the regular hospital-based clinic (which is connected to a rapid response team and the emergency department for additional support if required) or by referring physicians in outlying clinics. All patients seen on high-risk clinic days were included in the analysis, except those for whom procedures were not initiated. Excluded were patients who did not understand the purpose of their visit, patients who did not consent to the procedure, and patients with symptom flares needing management that were contraindications for proceeding with the procedure.

For each identified case in which an allergy procedure was performed on a high-risk procedure clinic day, we downloaded demographic data from the electronic medical record and manually reviewed each encounter to extract the type of procedure performed, the indications for the procedure, and the final procedure outcomes. The procedure outcomes included whether the procedure was completed, whether a systemic allergic reaction occurred, the nature of the reaction, and any treatment that was required. Each encounter was reviewed by 2 data extractors. When outcomes varied, a third reviewer evaluated the case to ensure accuracy of the data.

Data were used to generate descriptive statistics only for the purpose of establishing reaction rates for each procedure. No formal statistical analysis was conducted.

## RESULTS

During the span of 5.5 years and 66 clinic days, our high-risk allergy clinic performed a total of 596 procedures, averaging 9 procedures per clinic day. Patient demographics are presented in Table 1. Table 2 lists the types of procedures performed: 305 food challenges, 103 aeroallergen immunotherapy rush inductions, 75 drug challenges, 66 ultrarush inductions of venom immunotherapy, 12 drug desensitizations, 14 vaccine challenges (11 COVID-19 [coronavirus disease 2019], 2 influenza, 1 Tdap [tetanus, diphtheria, and pertussis]), and 21 miscellaneous nonvaccine procedures (pressure or cold challenge, latex challenge, perioperative anaphylaxis testing, radiocontrast media testing, penicillin skin testing, prick-to-prick with food, venom skin testing, hair dye skin tests, and topical medicine skin tests).

**Table 1.** Demographic Characteristics of Patients Presenting for High-Risk Allergy Procedures, n=596

Variable	Number of Patients
Pediatric (<18 years)	286 (48.0)
Adult (≥18 years)	310 (52.0)
Age	
Mean, years	25
Range	7 months to 86 years
Sex	
Female	277 (46.5)
Male	319 (53.5)
Race	
Caucasian or White	412 (69.1)
African American or Black	138 (23.2)
American Indian or Alaskan native	7 (1.2)
Asian	25 (4.2)
Other	5 (0.8)
Unknown	9 (1.5)

Note: Data are presented as n (%) unless otherwise indicated.

**Table 2.** Types of Challenges Performed, n=596

Challenge Type	Number
Food challenge	305 (51.2)
Aeroallergen immunotherapy rush induction	103 (17.3)
Drug challenge	75 (12.6)
Ultrarush induction of venom immunotherapy	66 (11.1)
Drug desensitization	12 (2.0)
Vaccine challenge (COVID-19, influenza, Tdap)	14 (2.3)
Miscellaneous nonvaccine challenges (pressure or cold challenge, latex challenge, perioperative anaphylaxis testing, radiocontrast media testing, penicillin skin testing, prick-to-prick with food, venom skin testing, hair dye skin tests, and topical medicine skin tests)	21 (3.5)

Note: Data are presented as n (%).

COVID-19, coronavirus disease 2019; Tdap, tetanus, diphtheria, and pertussis.

### Epinephrine Administration

Epinephrine was administered a total of 51 times, with 2 procedures resulting in emergency department transfers and none leading to patient mortality (Figure).

**Figure.**
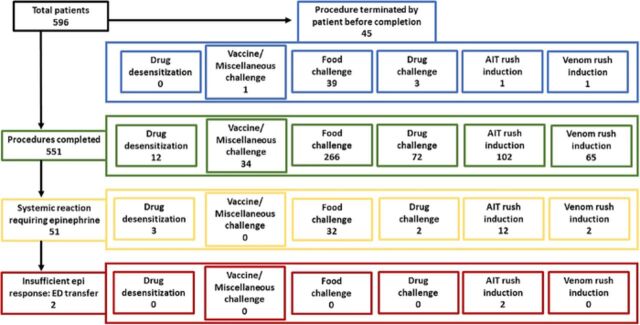
**Overview of procedures performed in the high-risk allergy procedure clinic**. AIT, aeroallergen immunotherapy; ED, emergency department; epi, epinephrine.

#### Food Challenge.

Thirty-two of the 305 (10.5%) food challenges resulted in epinephrine administration. Thirty-nine food challenges were terminated prior to completion by a patient or parent prior to a reaction occurring. Systemic reaction rates by food are presented in Table 3. The highest reaction rate was to the egg challenge (16.4%), followed by tree nut (12.0%), baked egg (11.9%), and shellfish (11.9%).

**Table 3.** Food Challenge Outcomes, n=305

	Food Challenge									
Outcome	Baked Egg, n=67	Egg, n=55	Baked Milk, n=17	Milk, n=17	Peanut, n=47	Fish, n=13	Shellfish, n=42	Tree Nut, n=25	Other, n=22	Total, n=305
Epinephrine administration	8 (11.9)	9 (16.4)	1 (5.9)	1 (5.9)	2 (4.3)	1 (7.7)	5 (11.9)	3 (12.0)	2 (9.1)	32 (10.5)
Ended early by patient or provider (no epinephrine)	12 (17.9)	10 (18.2)	2 (11.8)	4 (23.5)	7 (14.9)	1 (7.7)	0	2 (8.0)	1 (4.5)	39 (12.8)

Note: Data are presented as n (%).

Among the patients who passed an egg challenge, 9 of 36 (25.0%) had previously passed a baked egg challenge and consumed denatured egg for at least 4 months in an effort to achieve full egg tolerance. Similarly, 4 of the 14 (28.6%) patients who passed a milk challenge had previously passed a baked milk challenge and regularly consumed denatured milk proteins to achieve tolerance of all milk.

#### Aeroallergen Immunotherapy Rush Induction.

Our clinic employs 3 different aeroallergen immunotherapy rush induction protocols, each named according to the percentage of the final maintenance dose administered at the last stage of the protocol.^15^ These protocols are important because they determine the speed and concentration at which allergens are introduced, directly impacting the likelihood of allergic reactions. For instance, in the 10% protocol, the last injection delivers 10% of the total maintenance dose, whereas the 4% and 1% protocols introduce the allergen more gradually, with the final injection containing only 4% or 1% of the maintenance dose, respectively. This variation allows for tailoring the approach based on patient tolerance and safety considerations.

In our cohort, epinephrine was administered in 12 of 103 (11.7%) patients undergoing aeroallergen immunotherapy rush induction. Specifically, 9 of 52 (17.3%) patients who underwent the 10% protocol required epinephrine, compared to 3 of 31 (9.7%) patients who underwent the 4% protocol, and none of the 20 patients who underwent the 1% protocol. These data underscore the importance of selecting the appropriate protocol based on the individual patient's risk profile and reactivity.

#### Drug Challenge.

The 75 drug challenges consisted of 22 amoxicillin, 29 nonsteroidal anti-inflammatory drug (NSAID), 7 anesthetic, and 17 other drug challenges as shown in Table 4. Only 2 of 75 (2.7%) drug challenge procedures required epinephrine: 1 amoxicillin challenge in a patient who had a negative skin test and 1 NSAID challenge.

**Table 4.** Drug Challenge Outcomes, n=75

	Drug Challenge				
Outcome	Amoxicillin, n=22^a^	NSAID, n=29	Anesthetic, n=7	Other, n=17^b^	Total, n=75
Epinephrine administration	1 (4.5)	1 (3.4)	0	0	2 (2.7)
Ended early by patient (no epinephrine)	0	0	2 (28.6)^c^	1 (5.9)^d^	3 (4.0)

^a^Seventeen patients had a skin test; all were negative.

^b^The following drugs were challenged: 2 ranitidine, 1 azithromycin, 1 betamethasone, 1 prednisone, 1 orphenadrine, 3 cefdinir, 1 penicillin VK, 1 benzylpenicillin, 3 acetaminophen, 1 trimethoprim/sulfamethoxazole, 1 sublingual dust mite tablet, and 1 omalizumab.

^c^Both patients had the subjective sensation of their throat closing after the administration of lidocaine.

^d^The patient was challenged with cefdinir and experienced foot and trunk pruritus.

Note: Data are presented as n (%).

NSAID, nonsteroidal anti-inflammatory drug.

Three drug challenge procedures were aborted. Two challenges were stopped because the patients had the subjective sensation of their throat closing after administration of lidocaine: 1 after the intradermal for which distraction techniques successfully treated symptoms, and 1 after skin prick (the patient had a concurrent normal tryptase level). The third discontinuation occurred when a patient experienced pruritus after 7 mL of cefdinir and was given loratadine; the patient declined epinephrine and the symptoms resolved.

#### Ultrarush Induction of Venom Immunotherapy.

Our clinic serves as a referral center for patients with venom allergy; characteristics of these patient histories have been previously reported.^16^ Of the 66 venom desensitizations, 31 (47.0%) were to fire ant, 16 (24.2%) to paper wasp, 11 (16.7%) to honeybee, and 8 (12.1%) to mixed vespid. Of the 66 patients, 20 (30.3%) were <18 years old. Two patients (3.0%) received epinephrine: 1 during fire ant desensitization and the other during honeybee desensitization. One patient terminated the procedure prior to completion because of anxiety.

#### Drug Desensitization.

Of the 12 drug desensitization procedures, epinephrine was administered 3 times (25%). Notably, the 3 epinephrine administrations occurred in a single patient and were needed each time she underwent metronidazole desensitization. All other desensitizations were successful: 6 aspirin, 1 levothyroxine, 1 penicillin, and 1 metronidazole in a different patient. None of the drug desensitization procedures was aborted prior to completion.

#### Vaccine and Miscellaneous Nonvaccine Challenge.

Fourteen vaccine challenges and 21 miscellaneous nonvaccine challenge procedures were conducted: challenges with pressure (n=1), cold (n=1), latex (n=1), hand sanitizer (n=1), and bacitracin (n=1); skin testing for perioperative anaphylaxis (n=7), radiocontrast media (n=1), penicillin (n=2), hair dye (n=1), venom (n=1); and prick-to-prick with food (n=4). None of these procedures required epinephrine administration; 1 procedure was aborted early for psychosomatic symptoms.

## DISCUSSION

The implementation of a dedicated high-risk procedure clinic staffed by 1 allergist and 2 fellows is a strategic approach to concentrating patients with an elevated risk of anaphylaxis. This model not only allows for specialization within our group in performing high-risk allergy procedures but also ensures that allergy fellows gain valuable experience in diagnosing and managing anaphylaxis in the acute setting of a reaction.

### Psychosomatic Reactions

In tandem with the establishment of our high-risk clinic, we recognized an upswing in referrals for the evaluation of psychosomatic reactions. Patients with psychosomatic reactions often had a history of verified anaphylaxis to other triggers and expressed concerns about reactions to an increasing number of substances. Beyond anaphylaxis management, we have integrated approaches aimed at normalizing anxiety in individuals with past anaphylactic experiences that foster successful completion of challenges and successfully delabel medication and food allergies. Distraction techniques—such as discussing their loved ones or the hobbies and interests of either the provider or the patient—were often successful with these patients. We found that distraction helped redirect patients away from their focus on symptoms, which were often the subjective sensation of their throat closing or difficulty swallowing. A tryptase level can also be drawn shortly after the reaction to help address any underlying doubt of whether the reaction was an IgE-mediated reaction. The involvement of fellows in this process has not only contributed to improved patient care but also facilitated their skill development in managing psychosomatic reactions.

### Unexpected Benefits

An unforeseen advantageous outcome of our monthly high-risk clinic is that it has proven to be a safe and effective setting for managing urgent medication desensitizations in patients for whom inpatient admission for the procedure was not feasible. Our comfort in managing drug desensitizations in high-risk patients stemmed from the team's enhanced training in anaphylaxis management in this hospital-based clinic. Additionally, as COVID-19 immunizations became available—with minimal data available on systemic reactions—we were able to safely administer the vaccines to hospital staff who presented with risk factors such as urticaria following their first COVID-19 immunization. This early intervention in a controlled, high-risk setting was essential in providing necessary vaccinations while mitigating the risk of severe allergic reactions. Our experience mirrors what has been seen in other centers with no systemic allergic reactions to the COVID-19 immunizations.^17^

### Optimizing Food Challenges

A notable observation from our data is the number of uncompleted procedures, specifically food challenges terminated by young children before reaching the target amount. In response, we have adopted the approach of low-dose threshold food challenges which offer reassurance that patients who can consume a small quantity of the food are likely safe from accidental exposures.^18^ In retrospect, many of these incomplete procedures could be reframed as successful low-dose threshold challenges that we have since incorporated into practice.

### Strategic Procedure Scheduling

Based on these data showing that 8.6% of patients overall required epinephrine across all procedures, we have strategically adjusted our scheduling practices. Procedures now identified as lower risk (eg, 1% aeroallergen rush protocols and low-dose threshold food challenges) are now scheduled for alternate low-risk procedure days or on regular clinic days with the patients’ allergists. Procedures that are time consuming despite being low risk (eg, venom allergy and patients with a high probability of experiencing psychosomatic reactions) continue to be scheduled on our high-risk clinic days. This optimization ensures the efficient use of resources, preserving space in our high-risk clinic for patients with specific high-risk stratification and increasing access for patients who need lower risk procedures on alternate days.

### Study Limitations

Limitations of these data include the inability to estimate the true risk of these procedures without inclusion of similar procedures performed at other allergy clinics in our area. Patients referred to our high-risk clinic may have had a higher risk of reaction than those not referred; our data demonstrate a higher reaction rate than is seen in the general population. The risk estimates of infrequently performed procedures should be interpreted with caution, as the sampling error inherent with small sample sizes may overestimate or underestimate the true risks. Additionally, risk may vary geographically because of differences in exposure. For example, the New Orleans, Louisiana, area has low exposure to *Vespula* insects (called yellow jackets or wasps),^19^ making anaphylaxis to this venom type uncommon. Additionally, our high exposure to fire ants is inversely correlated with exposure to the ticks that trigger alpha-gal syndrome, making meat allergy uncommon in our area.^20^

## CONCLUSION

Allergists perform procedures that have variable levels of risk for triggering anaphylactic reactions. In health systems where patients are referred to high-risk centers, estimation of risk by referring physicians may be discordant with actual risk. We found that some procedures, including the 1% aeroallergen immunotherapy rush induction protocol, oral challenges with NSAIDs, and ultrarush desensitization with venom infrequently caused systemic allergic reactions, while food challenges, drug desensitizations, and the 10% aeroallergen immunotherapy rush induction protocol provoked higher rates of reaction and were treated with epinephrine. Benefits of having a high-risk allergy procedure clinic include the ability to quickly manage anaphylaxis, the time and experience to manage psychosomatic reactions, the ability to perform uncommonly performed procedures, and the training of fellows and residents in the management of anaphylaxis. Health systems where allergy procedures are performed should assess the risk of anaphylaxis to optimize the locations where these procedures are done.

## Notes

*This article meets the Accreditation Council for Graduate Medical Education and the American Board of Medical Specialties Maintenance of Certification competencies for Patient Care and Medical Knowledge.*
